# Construction and validation of a tracheostomy prediction model in mechanically ventilated stroke patients and the impact of early versus late tracheostomy on clinical outcomes: an IPTW-based analysis

**DOI:** 10.3389/fneur.2026.1853604

**Published:** 2026-07-21

**Authors:** Miaoxinhui Shao, Wenjie Pan, Yanxin Liu, Hui Wang, Xiaoxia Duan

**Affiliations:** 1 The Second Affiliated Hospital of Bengbu Medical University, Bengbu, China; 2 Department of Nursing, Bengbu Medical University, Bengbu, China; 3 School of Medical Imaging, Bengbu Medical University, Bengbu, China; 4 Department of Development and Cooperation, The Second Affiliated Hospital of Bengbu Medical University, Bengbu, China

**Keywords:** clinical outcomes, early tracheostomy, mechanically ventilated stroke, prediction model, tracheostomy

## Abstract

**Background:**

This study aimed to develop and validate a predictive model for tracheostomy in mechanically ventilated stroke patients and to investigate the impact of early and late tracheostomy on in-hospital outcomes.

**Methods:**

A total of 508 mechanically ventilated stroke patients who were admitted to a tertiary hospital between January 2022 and January 2025 were retrospectively enrolled and divided into the tracheostomy and non-tracheostomy groups. Patients were randomly split into a training set (*n* = 356) and a validation set (*n* = 152) at a ratio of 7:3. Least absolute shrinkage and selection operator (LASSO) regression combined with the importance of Random Forest feature was used to identify key variables. Independent predictors were identified using the multivariable logistic regression, and a nomogram was constructed. Model performance was evaluated using receiver operating characteristic (ROC) curves, calibration curves, and decision curve analysis (DCA). Inverse probability of treatment weighting (IPTW) based on propensity scores was applied to assess the effects of early (≤7 days) versus late (>7 days) tracheostomy on clinical outcomes.

**Results:**

A multivariable logistic regression identified midline shift, hypoalbuminemia, admission Glasgow Coma Scale (GCS) score, C-reactive protein (CRP), and prognostic nutritional index (PNI) as independent predictors (*p* < 0.05). The area under the curve (AUC) was 0.855 in the training set (sensitivity: 75.72%, specificity: 79.78%) and 0.849 in the validation set (sensitivity: 70.04%, specificity: 84.30%), indicating good discriminative ability. The Hosmer–Lemeshow test demonstrated good calibration (training set: *χ*^2^ = 6.943, *p* = 0.543; validation set: *χ*^2^ = 13.547, *p* = 0.094). DCA showed that the model provided a favorable net clinical benefit within a certain threshold range. IPTW analysis indicated that early tracheostomy significantly reduced ICU length of stay but had no significant effect on post-tracheostomy ventilation duration, antibiotic use duration, total hospital stay, or hospitalization costs.

**Conclusion:**

The nomogram developed in this study demonstrated good performance in predicting the risk of tracheostomy in mechanically ventilated stroke patients and enabled individualized real-time risk assessment via a web-based tool. Early tracheostomy may help shorten ICU stay and could inform clinical decision-making.

## 1 Introduction

Stroke, a major global public health challenge, has become the second leading cause of death and the third leading cause of disability worldwide (1). Data from the Global Burden of Disease (GBD) study (2) show that stroke causes approximately 7.3 million deaths annually. The incidence and disability burden remain on the rise in some regions, with a growing trend toward stroke occurring in younger adults under 55 years of age (3, 4). For critically ill stroke patients requiring prolonged mechanical ventilation support, tracheotomy is a commonly used and an important clinical intervention (5). Studies have reported that the tracheotomy rate is approximately 15% among general ICU patients, while it can be as high as 35% in critically ill stroke patients (6). Tracheotomy can reduce endotracheal intubation-related complications and improve patients’ ventilation tolerance and comfort. Tracheostomy is generally associated with a lower risk of ventilator-associated pneumonia (VAP) compared with long-term endotracheal intubation (7). Despite its significant benefits in improving ventilation function, tracheotomy may increase the risk of lower respiratory tract infections, thereby adversely affecting patients’ prognosis and rehabilitation (8). Previous studies have shown that only approximately one-third of stroke patients who undergo tracheotomy ultimately regain the ability to live independently (9). In addition, the timing of tracheotomy is a crucial decision point. Emerging evidence suggests that different timings of tracheotomy exert varying effects on patients with severe stroke, but no unified clinical consensus has been reached to date (10). Therefore, scientifically and accurately determining the optimal timing of tracheotomy and formulating individualized intervention strategies for mechanically ventilated stroke patients are of great significance for ensuring patient safety and improving long-term prognosis.

Predictive models have demonstrated tremendous potential in the field of stroke, particularly in disease prediction, subtype classification, risk factor identification, and risk stratification (11). The present study aims to construct and validate a tracheotomy risk prediction model specifically developed for mechanically ventilated stroke patients. Additionally, it intends to analyze the impact of early versus late tracheotomy on major in-hospital clinical outcomes of these patients, thereby providing a scientific basis for individualized clinical decision-making.

## 2 Materials and methods

### 2.1 Data source

A total of 508 mechanically ventilated stroke patients admitted to our hospital from January 2022 to January 2025 were enrolled in this retrospective study.

Inclusion criteria were as follows: (1) age ≥18 years; (2) diagnosis of stroke confirmed by magnetic resonance imaging (MRI) or computed tomography (CT); and (3) received mechanical ventilation support for >24 h.

Exclusion criteria were as follows: (1) incomplete clinical data; (2) tracheotomy performed prior to admission; (3) death within 24 h after admission; (4) comorbidity with malignant tumors, severe mental illnesses, or other end-stage critical diseases.

Patients who underwent tracheotomy were further divided into the early tracheotomy group, defined as tracheotomy performed within 7 days after endotracheal intubation, and the late tracheotomy group, defined as tracheotomy performed more than 7 days after endotracheal intubation, according to prior studies (10). Demographic data, laboratory parameters, disease- and treatment-related information, and in-hospital outcomes were collected from the electronic medical record system and independently reviewed by two researchers to ensure data accuracy. Missing or abnormal data were handled according to predefined criteria. This study was conducted in accordance with ethical principles and was approved by the Medical Ethics Committee of our hospital.

### 2.2 Variables

Clinical data of patients were collected from the electronic medical record system of our hospital, including: (1) general information: gender, age, and past medical history; (2) disease- and treatment-related indicators: stroke type, midline shift, baseline abnormal lung computed tomography (CT) findings (evaluated by X-ray/CT imaging upon admission or before tracheotomy, with records of pulmonary infiltration, atelectasis, or other abnormal imaging signs), Glasgow Coma Scale (GCS) score, hypoalbuminemia (albumin <30 g/L), ventilator-associated pneumonia (VAP), sedative and analgesic therapy, and history of craniocerebral surgery; and (3) laboratory indicators: red blood cells (RBC), white blood cells (WBC), neutrophils, lymphocytes, platelets, hemoglobin (Hb), albumin, and C-reactive protein (CRP). Additionally, composite inflammatory indicators were calculated, including platelet-to-lymphocyte ratio (PLR), neutrophil-to-lymphocyte ratio (NLR), lymphocyte-to-monocyte ratio (LMR), systemic immune-inflammation index (SII), and prognostic nutritional index (PNI). SII was calculated as platelet count × neutrophil count/lymphocyte count, and PNI was calculated as albumin (g/L) + 5 × lymphocyte count (10^9^/L). All predictive variables were derived from clinical and laboratory data collected within the first 24 h post-admission and before tracheotomy decision-making to ensure the inclusion of only baseline variables.

Furthermore, for patients who underwent tracheotomy, the duration of antibiotic use, post-tracheotomy ICU stay duration, the duration of mechanical ventilation after tracheotomy, total length of hospital stay, and total hospitalization costs were recorded.

### 2.3 Statistical methods

Statistical analyses were performed using SPSS 27.0 and R 4.4.2 software. Measurement data with a normal distribution were presented as the mean ± standard deviation, and comparisons between groups were conducted using the independent samples *t*-test. Measurement data with a non-normal distribution were expressed as median (interquartile range), and the Mann–Whitney *U* test was used for intergroup comparisons. Categorical data were described as frequencies and percentages, and the chi-square test was applied for intergroup comparisons.

This study was based on a single retrospective cohort; therefore, two analytic datasets were constructed according to different research objectives.

The first analysis aimed to develop a prediction model and was conducted using a complete-case analysis, including only tracheostomy patients with complete data on all key predictor variables. The dataset was randomly divided into a training set and a validation set at a 7:3 ratio; the training set was used for model construction, and the validation set for internal validation. Least absolute shrinkage and selection operator (LASSO) regression combined with Random Forest feature importance ranking was employed for variable selection, and the union of the selected variables was included in the multivariate logistic regression analysis to identify independent predictors and construct nomogram and dynamic nomogram models. The discriminative ability, calibration, and clinical benefit of the models were evaluated using the receiver operating characteristic (ROC) curve, calibration curve, and decision curve analysis (DCA).

The second analysis aimed to evaluate the association between the timing of tracheostomy and clinical outcomes. It included patients with complete information on the timing of tracheostomy and primary outcome variables, without requiring complete data for all baseline covariates. For patients undergoing early and late tracheotomy, inverse probability of treatment weighting (IPTW) was used to balance baseline confounders. Propensity scores were estimated using the multivariable logistic regression analysis, and stabilized weights were constructed with truncation to reduce the influence of extreme weights. Covariate balance was assessed using SMD, with an SMD of <0.1 indicating adequate balance. Propensity score overlap and weight distribution were also examined to assess common support and weighting stability.

## 3 Results

### 3.1 Characteristics of the study subjects

A total of 508 mechanically ventilated stroke patients were enrolled in this study, including 356 cases in the training set and 152 cases in the validation set. The baseline characteristics of the two groups were generally comparable (see Table 1). The results of the univariate analysis showed that there were statistically significant differences between the tracheotomy group and the non-tracheotomy group in terms of hypertension, stroke type, midline shift, admission GCS score, ventilator-associated pneumonia, sedative and analgesic therapy, hypoalbuminemia, decompressive craniectomy, other craniocerebral surgeries, C-reactive protein, hemoglobin, NLR, PLR, SII, and PNI (*p* < 0.05, see Table 2).

**Table 1:** Baseline characteristics between the training and validation sets.

Variables	Training set (*n* = 356)	Validation set (*n* = 152)	*t*/*χ*^2^/*Z*	*p-*value
Tracheotomy (%)	173 (48.6)	62 (40.8)	2.611	0.106
Age (years)	61.65 ± 12.405	62.74 ± 12.289	−0.903	0.367
Gender (male, %)	228 (64.0)	87 (57.2)	2.096	0.148
Hypertension (%)	282 (79.2)	125 (82.8)	0.852	0.356
Diabetes mellitus (%)	60 (16.9)	25(16.4)	0.013	0.910
Heart disease (%)	63 (17.7)	23 (15.1)	0.498	0.480
Previous stroke history (%)	83 (23.3)	34 (22.4)	0.054	0.817
Stroke type (%)			5.233	0.025
Ischemic stroke (%)	113 (31.7)	33 (21.7)		
Intracerebral hemorrhage (%)	243 (68.3)	119 (78.3)		
Baseline abnormal lung CT findings (%)	252 (70.8)	117 (77.0)	2.052	0.152
Midline shift (%)	114 (32.0)	41 (27.0)	1.281	0.258
Admission GCS score	8.82 ± 4.356	8.87 ± 4.057	−0.122	0.903
VAP (%)	327 (91.9)	144 (94.7)	1.311	0.252
Sedative and analgesic therapy (%)	215 (60.4)	85 (55.9)	0.881	0.348
Nasogastric tube placement (%)	79 (22.2)	34 (22.4)	0.002	0.965
Hypoalbuminemia (%)	124 (34.8)	47 (30.9)	0.729	0.393
Decompressive craniectomy (%)	114 (32.0)	56 (36.8)	1.111	0.292
Other craniocerebral surgeries (%)	221 (62.1)	107 (70.4)	3.220	0.073
CRP	50.52 (23.00–105.50)	56.60 (23.87–99.30)	−0.174	0.862
HB	103.00 (93.00–118.00)	103.00 (90.00–118.00)	−0.384	0.701
NLR	8.84 (6.06–13.76)	8.58 (5.89–13.40)	−0.641	0.522
PLR	218.18 (157.89–300.00)	199.50 (144.84–281.71)	−1.483	0.138
LMR	1.48 (1.06–2.07)	1.55 (1.09–2.22)	−0.639	0.523
SII	1769.71 (1146.02–2692.05)	1722.95 (995.99–2727.22)	−1.013	0.311
PNI	39.80 (36.10–43.50)	40.35 (36.43–44.76)	−1.209	0.227

GCS, Glasgow Coma Scale; VAP, ventilator-associated pneumonia; CRP, C-reactive protein; HB, hemoglobin; NLR, neutrophil-to-lymphocyte ratio; PLR, platelet-to-lymphocyte ratio; LMR, lymphocyte-to-monocyte ratio; SII, systemic immune-inflammation index; PNI, prognostic nutritional index.

**Table 2:** Clinical characteristics of patients in the training set.

Variables	Tracheotomy group (*n* = 173)	Non-tracheotomy group (*n* = 183)	*t*/*χ*^2^/*Z*	*p-*value
Age (years)	62.24 ± 12.39	61.10 ± 12.43	−0.870	0.385
Gender (male, %)	110 (63.6)	118 (64.5)	0.031	0.860
Hypertension (%)	147 (85.0)	135 (73.8)	6.776	0.009
Diabetes mellitus (%)	35 (20.2)	25 (13.7)	2.739	0.098
Heart disease (%)	33 (19.1)	30 (16.4)	0.439	0.508
Previous stroke history (%)	42 (24.3)	41 (22.4)	0.175	0.676
Stroke type (%)			5.100	0.024
Ischemic stroke (%)	45 (26.0)	68 (37.2)		
Intracerebral hemorrhage (%)	128 (74.0)	115 (62.8)		
Baseline abnormal lung CT findings (%)	126 (72.8)	126 (68.9)	0.681	0.409
Midline shift (%)	82 (47.4)	32 (17.5)	36.554	<0.001
Admission GCS score	7.25 ± 4.21	10.31 ± 3.96	7.055	<0.001
VAP (%)	168 (97.1)	159(86.9)	12.425	<0.001
Sedative and analgesic therapy (%)	116 (67.1)	99(54.1)	6.238	0.013
Nasogastric tube placement (%)	43 (24.9)	36 (19.7)	1.384	0.239
Hypoalbuminemia (%)	91 (52.6)	33 (18.0)	46.816	<0.001
Decompressive craniectomy (%)	67 (38.7)	47 (25.7)	6.952	0.008
Other craniocerebral surgeries (%)	118 (68.2)	103 (56.3)	5.371	0.020
CRP	80.65 (45.45–128.80)	31 (14.6–69.00)	−8.243	<0.001
HB	98.00 (87.00–115.00)	108.00 (96.00–120.00)	−4.339	<0.001
NLR	11.12 (7.23–17.92)	7.91 (5.27–10.37)	−6.090	<0.001
PLR	229.63 (164.15–328.74)	205.47 (150.78–274.77)	−2.566	0.010
LMR	1.41 (1.01–1.91)	1.56 (1.13–2.20)	1.873	0.024
SII	1981.55 (1213.88–3093.24)	1651.84 (1129.98–2321.37)	−2.264	0.003
PNI	37.75 (34.27–41.90)	41.92 (37.82–45.26)	−6.460	<0.001

### 3.2 Feature variable selection by LASSO regression and Random Forest importance ranking

**Figure 1:**
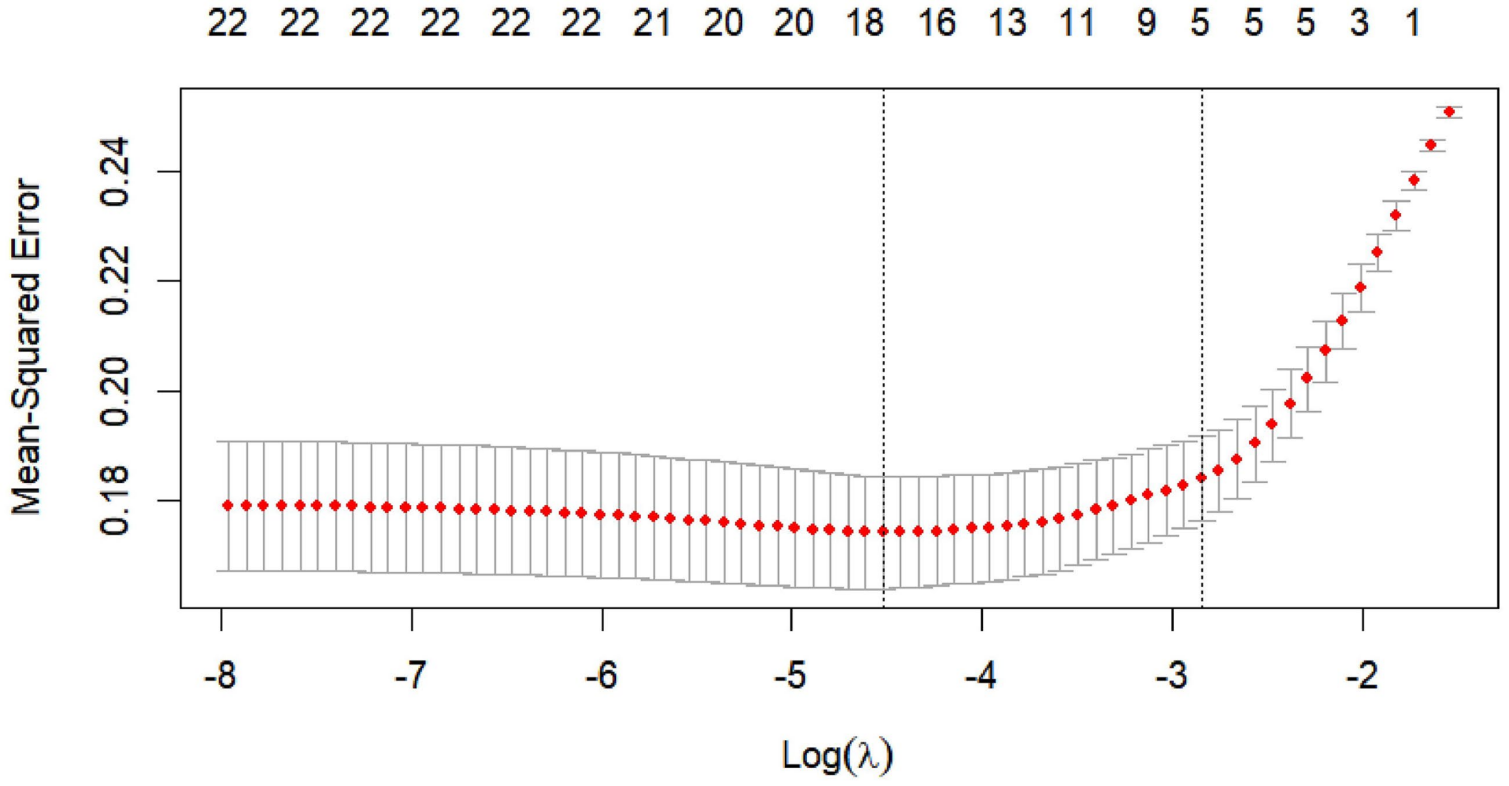
LASSO cross-validation curve.

**Figure 2:**
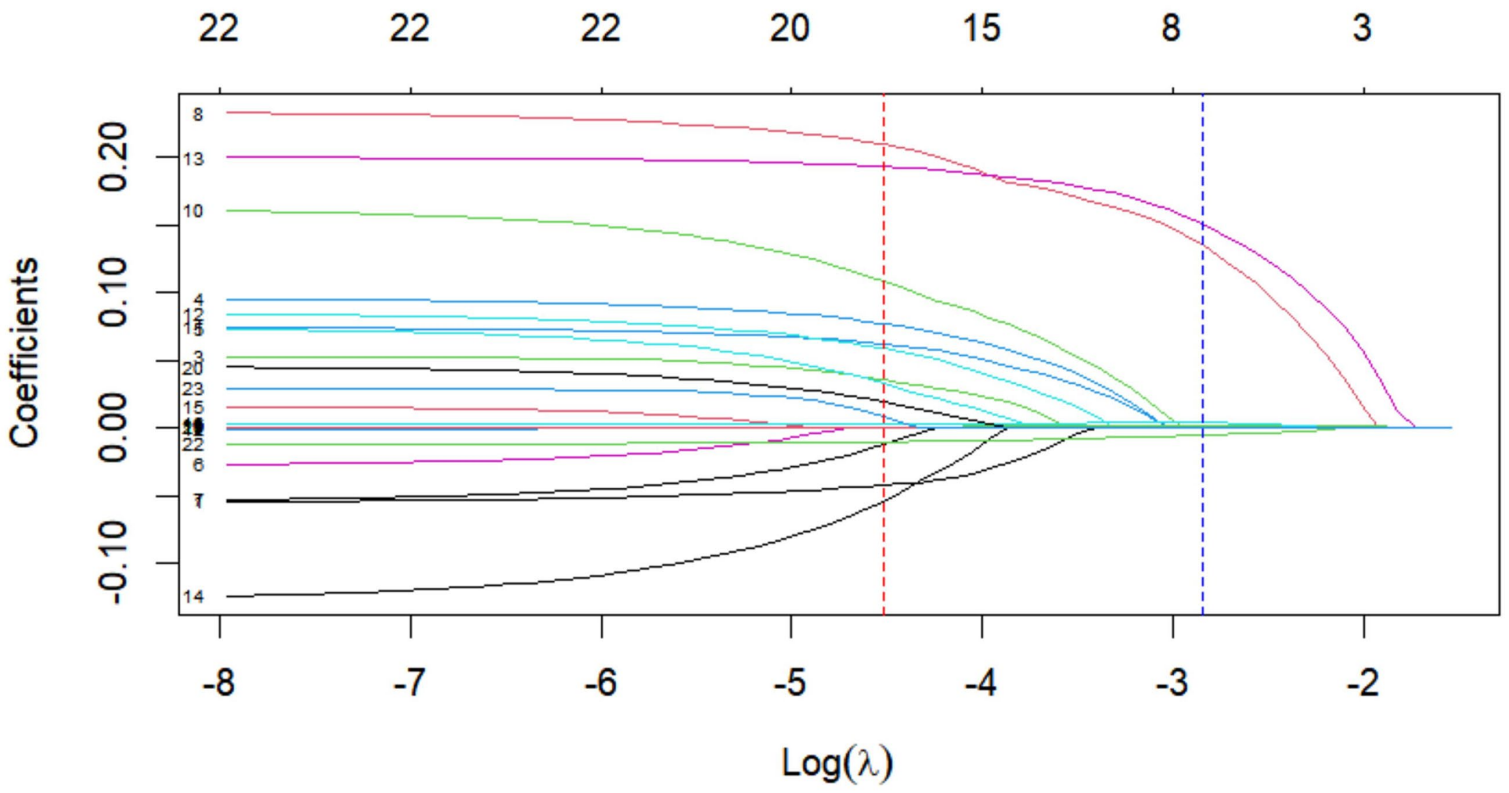
LASSO regression coefficient path plot.

**Figure 3:**
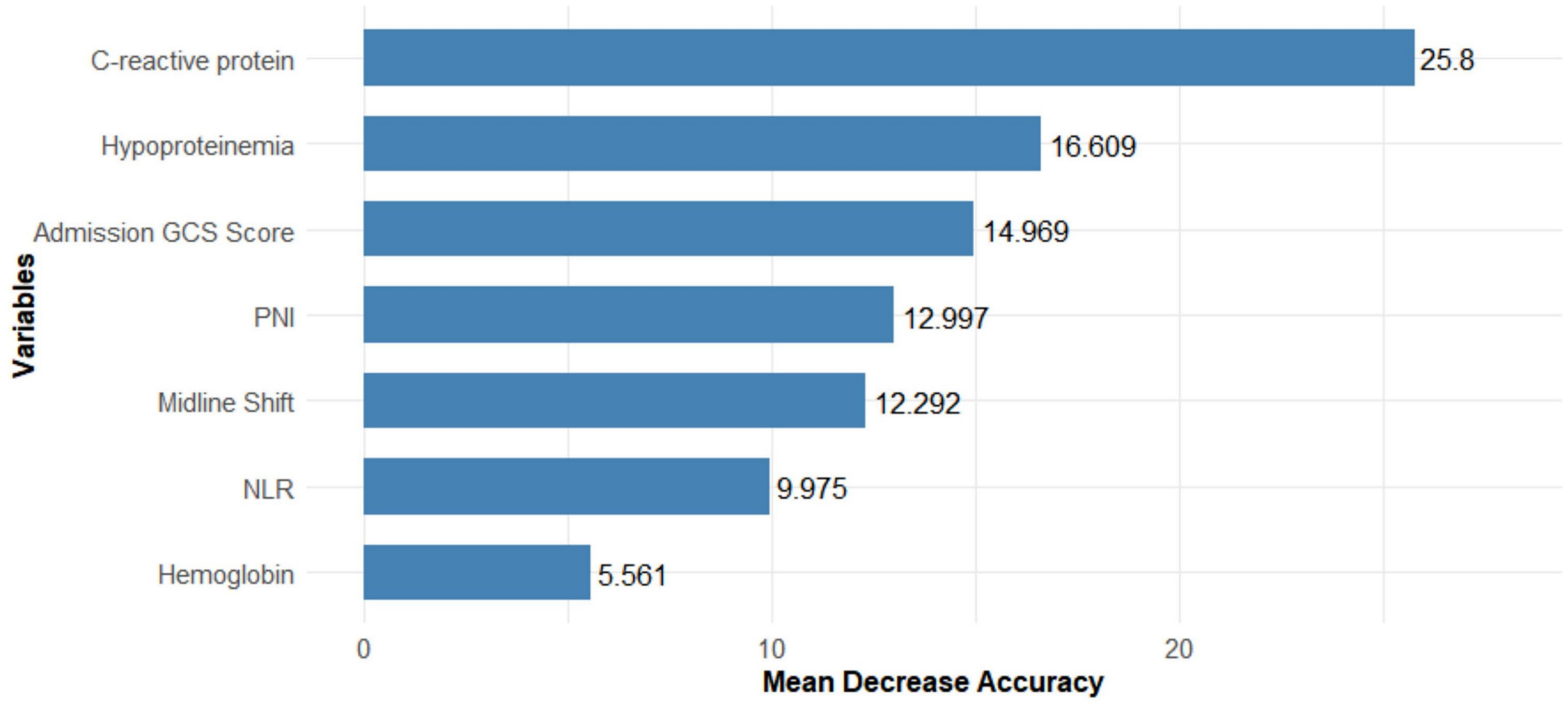
Random Forest importance ranking.

In this study, LASSO regression (L1 regularization) was used for sparse feature selection from all candidate variables. The optimal penalty coefficient within one standard deviation of the most parsimonious model (lambda.1se = 0.05823479) was determined via 10-fold cross-validation, leading to the identification of 5 significantly correlated variables: midline shift, hypoalbuminemia, CRP, NLR, and PNI (Figures 1, 2). Meanwhile, Random Forest feature importance ranking (mean threshold = 5.4444) was applied to evaluate variable contributions. The union of the selection results from the two methods was taken, and a total of seven variables—midline shift, hypoalbuminemia, CRP, NLR, PNI, admission GCS score, and Hb—were included in the initial multivariate logistic regression analysis (Figure 3). Preliminary fitting indicated that NLR exhibited high multicollinearity with other candidate variables, resulting in algorithm non-convergence. Further evaluation using the variance inflation factor (VIF) confirmed severe multicollinearity of NLR, which was thus excluded. The remaining six variables were retained for subsequent multivariate logistic regression modeling.

### 3.3 Multivariate logistic regression

After feature selection via LASSO regression and Random Forest, and exclusion of NLR with significant multicollinearity, midline shift, hypoalbuminemia, admission GCS score, Hb, CRP, and PNI were included in the multivariate logistic regression analysis. The results showed that midline shift, hypoalbuminemia, admission GCS score, CRP, and PNI were independent predictors of tracheotomy in mechanically ventilated stroke patients (see Table 3).

**Table 3:** Multivariate analysis results of predictive factors for tracheotomy in mechanically ventilated stroke patients.

Variables	Coefficients	Wald *χ*^2^	OR	95%CI	*p-*value	VIF
Midline shift	1.003	11.727	2.726	[1.536, 4.840]	<0.001	1.101
Hypoalbuminemia	1.328	21.098	3.772	[2.141, 6.646]	<0.001	1.100
Admission GCS score	−0.140	19.121	0.869	[0.816, 0.925]	<0.001	1.109
CRP	0.014	24.275	1.015	[1.009, 1.020]	<0.001	1.216
PNI	−0.076	8.990	0.927	[0.882, 0.974]	0.003	1.152

### 3.4 Model construction

**Figure 4:**
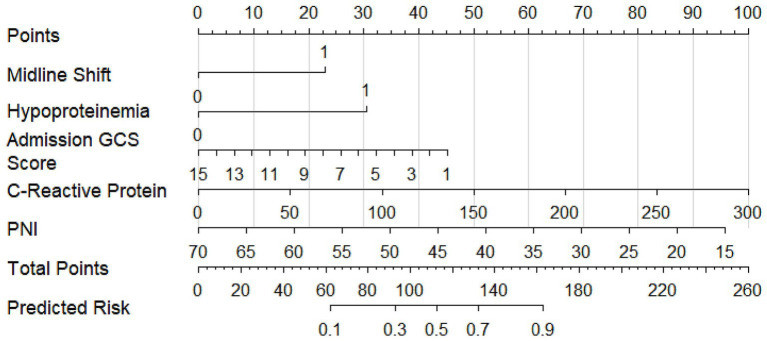
Nomogram model for tracheotomy in mechanically ventilated stroke patients.

**Figure 5:**
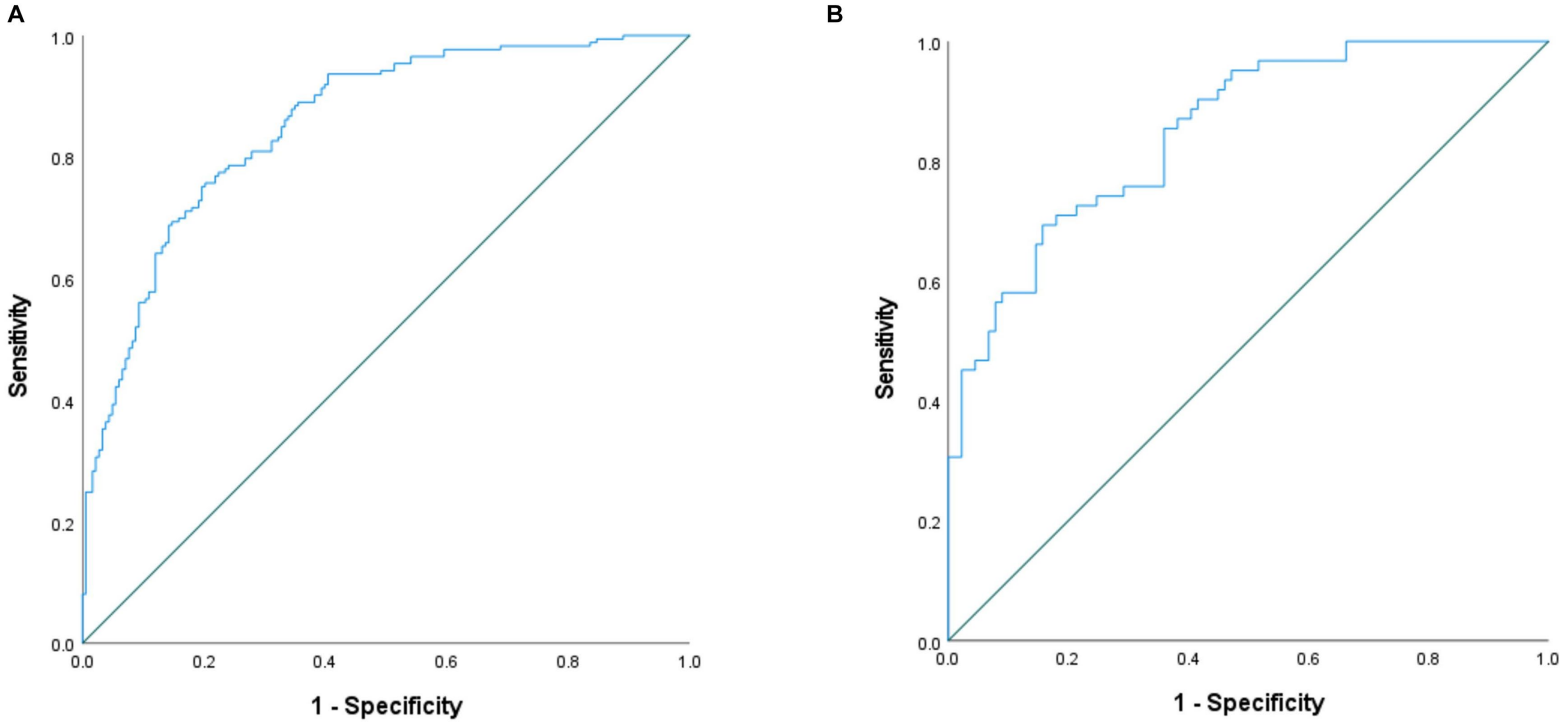
**(A)** ROC curve of the training set. **(B)** ROC curve of the validation set.

Based on the results of multivariate logistic regression analysis, a nomogram model was constructed to predict the risk of tracheotomy in mechanically ventilated stroke patients (Figure 4). Individualized assessment of tracheotomy risk in mechanically ventilated stroke patients can be achieved by reading the corresponding scores of each variable and calculating the total score. To further improve the clinical application value of the model, a dynamic nomogram model was developed using the R language Shiny package in this study (Figure 5). After users input the relevant clinical variables, the predicted probability can be automatically obtained and risk stratification can be realized (access link: https://stroke-tracheostomy-pred.shinyapps.io/stroke_app/).

### 3.5 Model evaluation and validation

**Figure 6:**
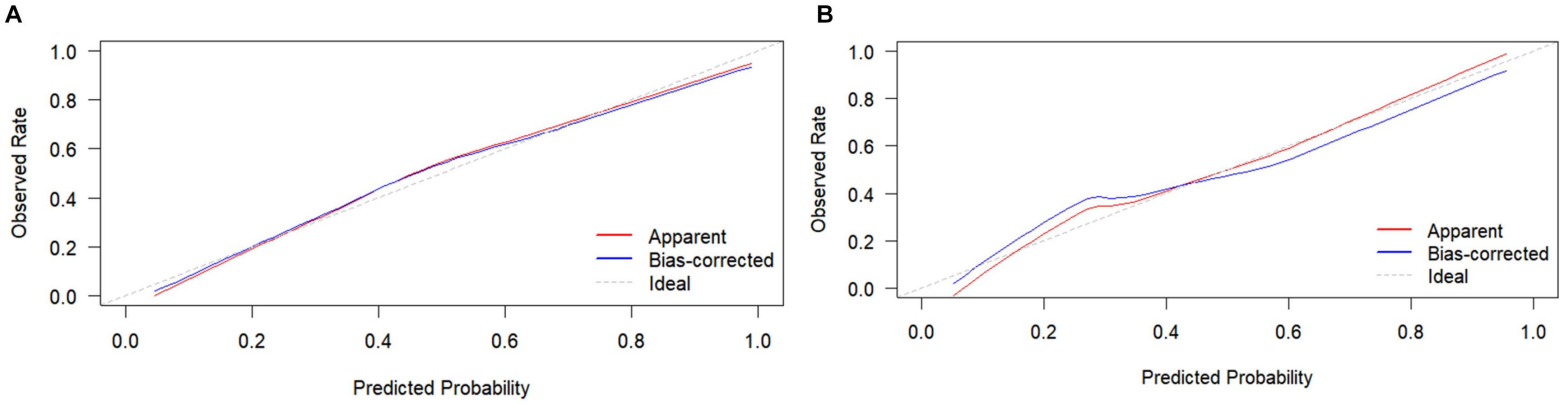
**(A)** Calibration curve of the training set. **(B)** Calibration curve of the validation set.

**Figure 7:**
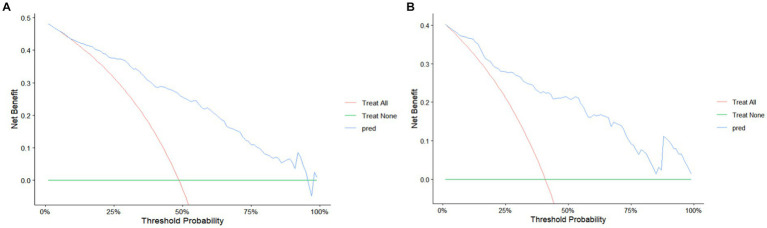
**(A)** Decision curve analysis of the training set. **(B)** Decision curve analysis of the validation set.

The model exhibited stable performance in both the training set and the internal validation set. In the training set, the AUC was 0.855 (95% CI: 0.816–0.893), with a sensitivity of 75.72% and a specificity of 79.78% (Figure 5A). After internal validation using bootstrap resampling, the optimism-corrected AUC was 0.848, with a calibration intercept of −0.025 and a calibration slope of 0.951. In the randomly split hold-out internal validation set, the model achieved an AUC of 0.849, with a sensitivity of 70.04%, a specificity of 84.30% (Figure 5B), an apparent calibration intercept of 0.000, and an apparent calibration slope of 1.150. Given that the ideal values of the calibration intercept and calibration slope are 0 and 1, respectively, these findings indicated good discrimination and overall calibration performance of the model. The calibration curves were close to the ideal line, and the Hosmer–Lemeshow test further supported acceptable calibration in both the training set and the validation set (training set: *χ*^2^ = 6.943, *p* = 0.543; validation set: *χ*^2^ = 13.547, *p* = 0.094) (Figures 6A,B). DCA demonstrated that the model had potential clinical application value across different threshold probabilities (Figures 7A,B).

### 3.6 Comparison of baseline characteristics between patients undergoing early and late tracheotomy before and after IPTW weighting

This analysis included 269 patients with complete information on tracheostomy timing and primary outcomes (Table 4), which differed from the population used in Table 1 (*n* = 235). This difference was due to the use of a complete-case dataset for prediction model development shown in Table 1. Although both analyses were derived from the same original cohort, differences in study objectives and inclusion criteria resulted in distinct analytic subsets.

**Table 4:** Comparison of baseline characteristics between early and late tracheotomy patients before and after IPTW weighting.

Variables	Early tracheotomy group (*n* = 163)	Late tracheotomy group (*n* = 106)	*t*/*χ*^2^/*Z*	*p-*value	
				Before IPTW weighting	After IPTW weighting
Age (years)	60.76 ± 13.18	64.58 ± 11.34	−2.448	0.015	0.696
Gender (male, %)	107 (65.6)	64 (60.4)	0.769	0.380	0.442
Hypertension (%)	143 (87.7)	83 (78.3)	4.251	0.039	0.476
Diabetes mellitus (%)	33 (20.2)	18 (17.0)	0.445	0.505	0.893
Heart disease (%)	30 (18.4)	19 (17.9)	1.548	0.461	0.603
Previous stroke history (%)	40 (24.5)	28 (26.4)	0.120	0.729	0.884
Stroke type (%)			0.580	0.446	0.949
Ischemic stroke (%)	42 (25.8)	23 (21.7)			
Intracerebral hemorrhage (%)	121 (74.2)	83 (78.3)			
Baseline abnormal lung CT findings (%)	125 (76.7)	68 (64.2)	4.980	0.026	0.927
Midline shift (%)	77 (47.2)	51 (48.1)	0.020	0.888	0.727
Admission GCS score	6.80 ± 3.92	7.73 ± 4.40	−1.810	0.071	0.709
VAP (%)	161 (99.4)	101 (95.3)	4.920	0.027	0.992
Sedative and analgesic therapy (%)	105 (64.8)	69 (65.1)	0.002	0.963	0.790
Nasogastric tube placement (%)	29 (17.8)	32 (30.2)	5.630	0.018	0.980
Hypoalbuminemia (%)	85 (52.1)	53 (50.0)	0.119	0.731	0.379
Decompressive craniectomy (%)	68 (41.7)	47 (44.3)	0.180	0.671	0.995
Other craniocerebral surgeries (%)	110 (67.5)	81 (76.4)	2.488	0.115	0.932
CRP	100.76 (53.60–141.30)	63.05 (34.58–100.50)	−4.023	<0.001	0.224
HB	100.00 (88.00–116.00)	94.00 (84.00–104.25)	−2.735	0.006	0.333
NLR	10.32 (7.00–16.01)	10.67 (7.31–17.64)	−0.709	0.478	0.977
PLR	224.56 (157.69–312.50)	245.52 (177.03–353.44)	−1.681	0.093	0.474
LMR	1.45 (1.01–1.91)	1.47 (1.03–2.23)	−1.091	0.275	0.822
SII	1738.24 (1072.42–2612.52)	2611.17 (1292.25–4206.29)	−3.798	<0.001	0.433
PNI	36.45 (33.95–41.30)	39.97 (36.28–42.25)	−3.432	0.012	0.413

**Figure 8:**
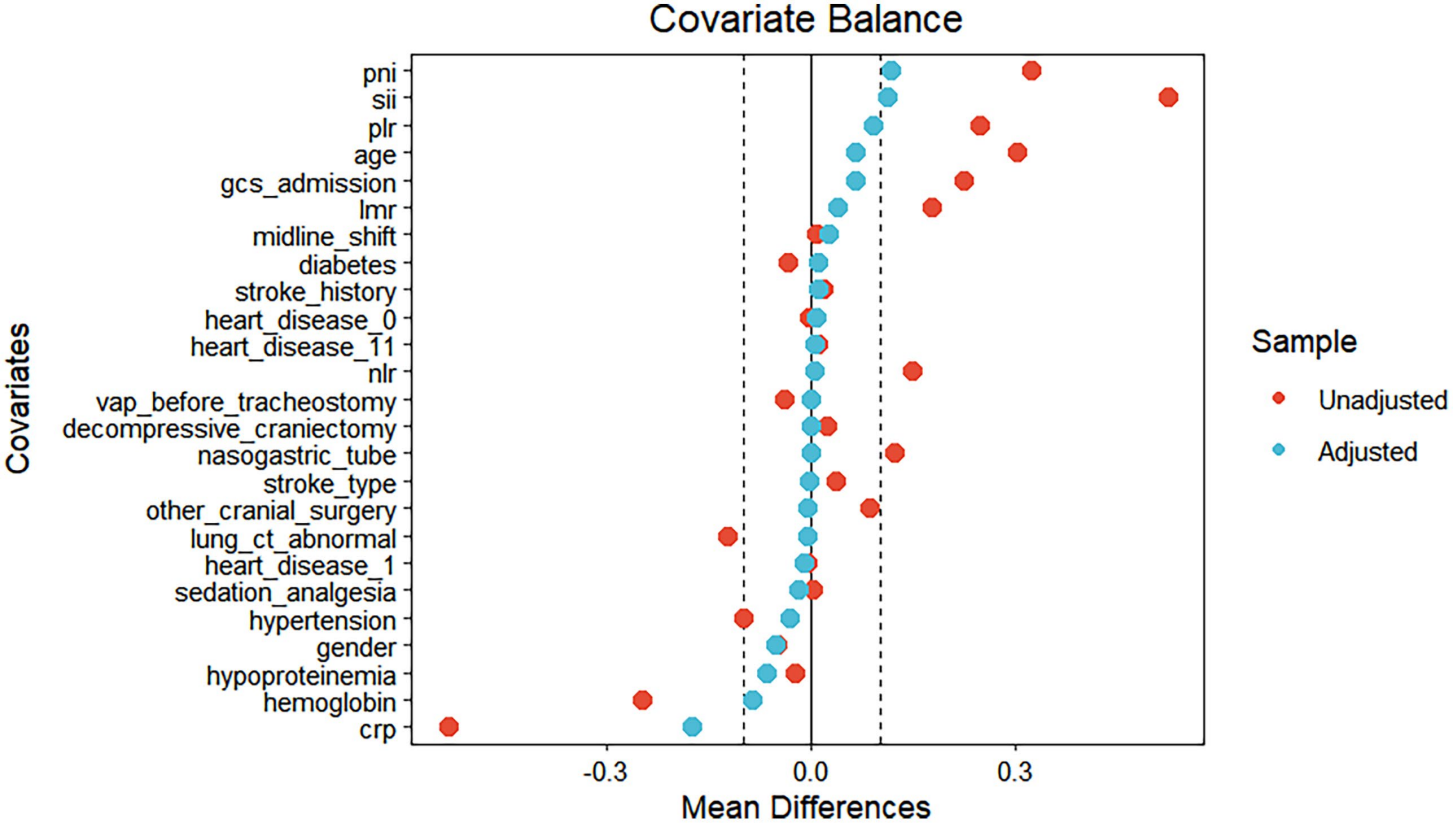
Changes in standardized mean difference (SMD) of each covariate before and after IPTW weighting.

Before IPTW was performed, there were significant differences in variables between the two tracheotomy groups with respect to age, hypertension, baseline abnormal lung CT findings, VAP, nasogastric tube placement, CRP, Hb, SII, and PNI (*p* < 0.05, see Table 4). After IPTW weighting, the SMD of each covariate was significantly reduced, with most decreasing to below 0.1 and all converging toward 0. This indicated that the baseline characteristics of the two groups had basically achieved comparability (Figure 8). The two groups showed adequate propensity score overlap, and no obvious extreme weights were observed after truncation, supporting the reliability of the weighted analysis (Supplementary Figure 1).

### 3.7 Comparison of clinical outcomes between patients undergoing early and late tracheotomy after IPTW weighting

After IPTW weighting, the length of ICU stay in the early tracheotomy group was significantly shorter than that in the late tracheotomy group, with a statistically significant difference (*p <* 0.001). However, no statistically significant differences were observed in the duration of mechanical ventilation after tracheotomy, the duration of antibiotic use, total length of hospital stay, or total hospitalization costs between the two groups (*p* > 0.05, see Table 5).

**Table 5:** Comparison of clinical outcomes between early and late tracheotomy patients after IPTW weighting.

Variables	Early tracheotomy group (*n* = 163)	Late tracheotomy group (*n* = 106)	*t*/*χ*^2^/*Z*	*p-*value	
				Before IPTW weighting	After IPTW weighting
Duration of antibiotic use (days)	23.00 (15.00–31.00)	25.00 (18.00–38.00)	−1.955	0.051	0.191
Length of ICU stay (days)	13.00 (10.00–16.00)	16.00 (12.75–20.00)	−5.630	<0.001	<0.001
Duration of mechanical ventilation after tracheotomy (days)	21.00 (12.00–30.00)	19.00 (11.00–30.00)	−0.627	0.531	0.520
Total length of hospital stay (days)	29.00 (20.00–40.00)	33.50 (22.00–47.25)	−2.245	0.025	0.112
Total hospitalization costs (yuan)	129657.73 (93631.43–193151.24)	153247.85 (103671.87–228223.73)	−2.785	0.018	0.266

## 4 Discussion

This study identified midline shift, hypoalbuminemia, admission GCS score, CRP, and PNI as independent predictors of tracheotomy in mechanically ventilated stroke patients. A risk prediction model was constructed based on the aforementioned indicators. The model exhibited favorable discriminative performance and calibration in both the training set and the validation set, which can be utilized for the early identification of mechanically ventilated stroke patients at high risk of requiring post-stroke tracheotomy and providing a reference for clinical decision-making.

We found that a lower admission GCS score was associated with a higher risk of tracheotomy in mechanically ventilated stroke patients. This conclusion was supported by previous studies. For instance, Yaghi et al. (12) reported that an admission GCS score of 8 was significantly correlated with the need for tracheotomy in patients with intracerebral hemorrhage and severe stroke. Similarly, Birtane et al. (13) observed that a low GCS score was a key determinant of tracheotomy in critically ill mechanically ventilated patients, further validating the findings of the present study. In addition, a low GCS score has been confirmed to be closely associated with an increased risk of weaning failure and in-hospital mortality in mechanically ventilated patients (14). This may be attributed to the fact that patients with low GCS scores suffer from severe disturbance of consciousness, weakened cough reflex, and impaired airway protection ability, which predispose them to pulmonary infection and airway obstruction. Collectively, these observations further suggest that timely airway intervention is warranted for patients with a low admission GCS score.

We also found that midline shift was an important independent risk factor for tracheotomy in mechanically ventilated stroke patients. Midline shift usually reflects the degree of intracranial mass effect and brain tissue compression. Such patients are often accompanied by elevated intracranial pressure (ICP); sustained elevation of ICP can lead to brainstem compression and dysfunction, impair the regulatory function of the respiratory center, weaken airway protective reflexes, and induce a series of respiratory-related complications (e.g., neurogenic pulmonary edema), thereby increasing patients’ dependence on mechanical ventilation (15). Previous studies have confirmed that midline shift is closely associated with poor neurological outcomes in patients (16), particularly, those with significant midline shift in the early stage of onset are more prone to aggravated disturbance of consciousness and neurological deterioration. Aggravated disturbance of consciousness further impairs airway protection ability, ultimately increasing the need for tracheotomy.

Patients with elevated CRP levels were more likely to undergo tracheotomy, as this indicator reflects the degree of systemic inflammatory response. Following stroke onset, the body activates microglia to secrete interleukin-6 (IL-6), which stimulates the liver to produce CRP. This inflammatory response not only disrupts the blood–brain barrier (BBB) and exacerbates cerebral edema and neurological damage (17) but also may further aggravate brain tissue injury by activating the secondary complement system (18). In addition, elevated serum CRP levels in a majority of the stroke patients indicate an increased risk of pulmonary infection and respiratory dysfunction, which may further augment the need for tracheotomy. Herold et al. (19) have confirmed that CRP serves as a robust inflammatory marker for predicting the need for mechanical ventilation in patients: those with elevated CRP levels had a significantly higher mechanical ventilation rate than those with normal CRP levels. The increased demand for mechanical ventilation further elevates the risk of tracheotomy, which is highly consistent with the findings of the present study.

Both hypoalbuminemia and decreased PNI were associated with an increased risk of tracheotomy in mechanically ventilated stroke patients, and both indicators can reflect the nutritional and immune status of patients. Critically ill patients are often in a state of hypercatabolism and are frequently accompanied by diaphragmatic dysfunction. Without adequate nutritional support, they are prone to malnutrition, which, in turn, impairs immune function and respiratory muscle performance, thereby increasing the risk of adverse outcomes (20). Hypoalbuminemia not only exacerbates systemic inflammation and acute lung injury and elevates the risk of mortality but also serves as an important indicator of poor prognosis in various diseases. It can lead to a vicious cycle of disease progression, delay recovery, and increase the probability of tracheotomy (21). A decrease in PNI usually indicates hypoalbuminemia and lymphopenia, reflecting that the body is in a state of malnutrition and immunosuppression. This state can weaken the patient’s anti-infective capacity, increase the risk of complications such as pulmonary infection, prolong the duration of mechanical ventilation, and thus raise the likelihood of tracheotomy (22).

In addition, among stroke patients who underwent tracheotomy, after balancing baseline confounding factors using IPTW, early tracheotomy was associated with a shorter ICU stay compared with late tracheotomy. However, no significant differences were observed between the two groups in the duration of mechanical ventilation after tracheotomy, the duration of antibiotic use, total length of hospital stay, or total hospitalization costs. Several differences observed before weighting were attenuated after IPTW adjustment, suggesting that these findings may have been partly influenced by baseline imbalances and confounding factors. This finding is partly consistent with previous studies showing that early tracheotomy may be associated with a shorter ICU stay but may not provide clear advantages in reducing mechanical ventilation duration or hospitalization costs (23). However, another meta-analysis reported no significant association between early tracheotomy and ICU stay or total hospital stay (24). These inconsistent findings may be related to differences in study populations, disease severity, definitions of early tracheotomy, and adjustment for baseline confounders. Therefore, our results should be interpreted cautiously. For critically ill mechanically ventilated stroke patients, early tracheotomy may be associated with reduced ICU stay, but its potential benefit for other clinical outcomes appears limited. Individualized decision-making based on neurological status, respiratory condition, disease severity, and expected prognosis remains necessary in clinical practice.

This prediction model is primarily applicable to stroke patients who require mechanical ventilation for more than 24 h during the early stage of hospitalization and may provide a quantitative reference for tracheotomy-related clinical decision-making, thereby supporting individualized patient management. However, given the selective nature of the study population and the lack of stratified analyses for ischemic and hemorrhagic stroke, the applicability of the model to other patient populations and different stroke subtypes requires further validation. In addition, this study primarily focused on in-hospital outcomes and lacked follow-up data on long-term functional recovery and mortality. Future multicenter, large-scale prospective studies are warranted to further evaluate the generalizability and clinical utility of this model.

## 5 Conclusion

In this study, a risk prediction model for tracheotomy in mechanically ventilated stroke patients was constructed and validated, which can be used to identify high-risk patients early and assist in clinical decision-making. Our data suggested a potential association between early tracheotomy and reduced ICU stay duration; however, its impact on other clinical outcomes requires further validation.

## Data Availability

The datasets presented in this article are not readily available because the dataset contains potentially sensitive patient information, including clinical characteristics and outcomes. Due to institutional data protection policies and patient privacy regulations, the data cannot be made publicly available. However, de-identified data may be shared upon reasonable request to the corresponding author, subject to approval from the ethics committee of the Second Affiliated Hospital of Bengbu Medical College. Requests to access the datasets should be directed to 3254164014@qq.com.
